# Non-suicidal self-injury and suicide attempt: A continuum or separated identities?

**DOI:** 10.1192/j.eurpsy.2021.463

**Published:** 2021-08-13

**Authors:** L. Cammisa, S. Pacifici, D. Alunni Fegatelli, D. Calderoni, F. Fantini, M. Ferrara, A. Terrinoni

**Affiliations:** 1 Department Of Human Neuroscience, Section Of Child And Adolescent Neuropsychiatry, Sapienza University of Rome, Rome, Italy; 2 Public Health And Infectious Diseases, Sapienza University of Rome, Rome, Italy

**Keywords:** Suicide Attempt, adolescence, non-suicidal self-injury, Suicidality

## Abstract

**Introduction:**

Non-suicidal self-injury (NSSI) has been proposed as diagnostic entity and was added in the section 3 of the DSM 5. However, little is known about the long-term course of the disorder: NSSI and suicide attempt (SA) often lie on a continuum of self-harm, but it’s still unclear if they represent two different nosografical entities. Both these groups are commonly enclosed in the term of Deliberate self-harm (DSH), also including self-harm with suicidal intent conditions.

**Objectives:**

This study aims to explore differences between two clinical samples (NSSI and SA) to highlight the possible connection between these two categories, to better understand the risk of progression from NNSI into suicidal intent conditions.

**Methods:**

102 inpatients with DSH (62 NNSI; 40 SA; age range: 12 to 18 years) were assessed by self-report questionnaires: the Deliberate Self-Harm Inventory (DSHI) and the Repetitive Non-suicidal Self-Injury Questionnaire (R-NSSI-Q) to explore the severity and repetitiveness of self-injurious behaviors and by the Beck Hopelessness Scale (BHS) and Multi-Attitude Suicide Tendency scale (MAST), as indirect measures of suicidal risk.

**Results:**

Preliminary results showed that inpatients with NSSI (62) presented high scores of indirect suicide risk, similar to SA sample (40).

**Conclusions:**

This result highlights the possibility to consider NSSI and SA in a continuum of psychopathology and that repetitive self-harm even in the absence of clear suicidal intentions represent a significant risk factor in the development of suicidality in adolescence.

**Disclosure:**

No significant relationships.

